# C-reactive protein concentrations are higher in dogs with stage IV chronic kidney disease treated with intermittent hemodialysis

**DOI:** 10.1371/journal.pone.0274510

**Published:** 2022-09-22

**Authors:** Maria Gabriela Picelli de Azevedo, Silvano Salgueiro Geraldes, Paula Bilbau Sant’Anna, Beatriz Poloni Batista, Suellen Rodrigues Maia, Reiner Silveira de Moraes, Elizabeth Moreira dos Santos Schmidt, Fabiana Ferreira de Souza, Alessandra Melchert, João Carlos Pinheiro Ferreira, Carmel Rezende Dadalto, Henry David Mogollón García, Priscylla Tatiana Chalfun Guimarães-Okamoto

**Affiliations:** 1 Department of Veterinary Clinics, School of Veterinary Medicine and Animal Science, São Paulo State University (UNESP), Botucatu, São Paulo, Brazil; 2 São Caetano do Sul, São Paulo, São Paulo, Brazil; 3 Pet Care Veterinary Hospital Pacaembu Unit, São Paulo, São Paulo, Brazil; 4 Department of Veterinary Surgery and Animal Reproduction, School of Veterinary Medicine and Animal Science, São Paulo State University (UNESP), Botucatu, São Paulo, Brazil; The University of the West Indies, JAMAICA

## Abstract

In chronic kidney disease dogs, the inflammatory process increases C-reactive protein concentrations. This study aimed to determine C-reactive protein serum concentrations in stage IV chronic kidney disease dogs treated with intermittent hemodialysis. A prospective cohort study was conducted with 23 dogs allocated into three groups: control group (CG, n = 7), intermittent hemodialysis group (IHG, n = 8) and clinical treatment group (CTG, n = 8), both comprised of stage IV chronic kidney disease dogs. One blood sample from CG (initial evaluation) and two samples from IHG and CTG (first- and last-moment) were obtained to determine C-reactive protein concentration, total leukocytes, platelets, erythrocytes, total plasma protein, serum albumin, urea, creatinine, and phosphorus. C-reactive protein was higher in IHG compared to CG in the first- and last-moments (p <0.001) and compared to CTG in the first-moment (p = 0.0406). C-reactive protein presented moderate positive correlation with leukocytes (r = 0.5479; p = 0.01), and moderate negative correlation with albumin (r = - 0.5974; p = 0.006) and red blood cells (r = - 0.5878, p = 0.01). A high correlation coefficient was observed in the tests’ evaluation (CI = 0.59–0.78; r = 0.70; P<0.0001). In conclusion, both assays used in this study to measure C-reactive protein have provided safe and reliable quantification of the results. Additionally, despite IHG dogs presented an active inflammatory profile, intermittent hemodialysis has proven to be beneficial, leading to a clinical improvement in life quality of patients, and thus being recommended for stage IV CKD dogs when performed by trained professionals.

## Introduction

The chronic kidney disease (CKD) may affect dogs regardless of age, even though it is more commonly observed in elderly animals. The diagnosis is made through the determination of kidney disease biomarkers concentration that indicate a reduction in the glomerular filtration rate [1]. According to the International Renal Interest Society, CKD is classified in four stages based on the serum concentrations of creatinine and symmetric dimethylarginine, with substaging based on the urinary protein to creatinine ratio and the systolic blood pressure [2]. Stage IV of CKD is marked by severe azotemia, serum creatinine exceeding 5 mg/dL and symmetric dimethylarginine ≥ 54 μg/dL. Thus, renal replacement therapies are indicated at this stage if the animal remains unresponsive to the conventional clinical treatment [2].

Intermittent hemodialysis is an extracorporeal renal replacement therapy used in humans to treat patients with CKD. In veterinary medicine, the therapy is indicated for CKD patients in uremic crisis or in stage IV CKD [3,4] and acute kidney injury [5].

The use of stem cells and the possible immunomodulatory properties in patients with naturally occurring CKD has been investigated. Preliminary data in cats revealed a reduction in serum creatinine values and an increase in GFR, however, studies with naturally occurring CKD in dogs are needed [6,7].

CKD patients present a chronic inflammation profile due to systemic alterations caused by the disease [8], which have been recognized as a condition that promotes clinical manifestations of uremic crisis [9]. Therefore, inflammatory process may be associated with morbidity and mortality rates observed in patients affected by the disease [8]. Moreover, the clinical condition is intensified by the magnitude and prevalence of systemic inflammation [8]. The acute phase response is part of the organism immune defense when an inflammatory stimulus [10] such as infection, inflammation, surgical trauma or stress [11]. During the acute phase response, the organism releases proinflammatory cytokines such as interleukin-1, interleukin-6 and tumor necrosis factor alpha [12]. These cytokines stimulate the synthesis and release of acute phase proteins by the liver [12] and other organs [13].

Acute phase proteins play a role in restoring homeostasis and restraining microbial growth before adaptive immune response development [14]. In dogs, C-reactive protein is classified as a major positive acute phase protein and albumin as a negative acute phase protein [15]. In humans with CKD, 25% of the patients presented high C-reactive protein concentrations during hemodialysis sessions [16]. In dogs, increased C-reactive protein concentrations were reported in association with several diseases [17], including viral infection [18] and CKD [19].

Despite intermittent hemodialysis technique may cause an inflammatory reaction as the removal of undesirable solutes and the control of hydroelectrolytes unbalance in dogs with CKD is performed, it is hypothesized that it is also able to promote a clinical improvement in the animals’ quality of life. Hence, this study aimed to evaluate the inflammatory profile through the determination of C-reactive protein concentration of dogs in stage IV CKD treated with intermittent hemodialysis. The evaluation was conducted by two different methods. Besides the inflammatory profile observed in animals undergoing intermittent hemodialysis, the therapy has proven to be beneficial, improving clinical condition and life quality of the animals.

## Materials and methods

This study was approved by the Animal Use Ethics Committee (CEUA) from School of Veterinary Medicine and Animal Science, UNESP, (protocol number 0215/2017). Written informed consent was obtained from all dog owners.

### Group selection

In this study, the animals were allocated into three groups: control group (CG), intermittent hemodialysis group (IHG) and clinical treatment group (CTG). The CG comprehended 7 owned dogs, clinically healthy which were randomly selected regardless of gender or breed, weighing from 10 to 35 kg. In the CG, clinically healthy animals were considered as animals with the absence of alterations in complete blood count, serum biochemistry, urinalysis, abdominal organs during ultrasound evaluation, or cardiorespiratory, cutaneous, gastrointestinal, ocular or locomotor systems. Also, animals in convalescence period following any disease or surgical procedure six months before were excluded from the CG.

Both IHG and CTG comprised of 8 owned dogs diagnosed with stage IV CKD, which were randomly allocated. Similarly, the dogs were selected regardless of gender, age or breed, weighting from 10 to 35 kg. In these groups, the diagnose relied on serum creatinine (> 5 mg/dL) and ultrasound alteration compatible with CKD (small and irregular kidneys, increased echogenicity, decrease or lack of corticomedullary differentiation) [2,20]. For IHG and CTG groups, animals with acute kidney injury over the course of CKD, nephrolithiasis or kidney neoplasms, shock, clotting disorders, heart failure, signs of bacterial infection in the leukogram, and pancreatitis were excluded.

### Clinical treatment

The clinical treatment included intravenous fluid therapy with Ringer’s lactate. Emesis was controlled with ondansetron (0.5–1 mg/kg IV, BID or TID) or maropitant citrate (1 mg/Kg SC, SID); and gastric acid secretion with omeprazole (0.7 mg/Kg, IV, SID). The use of human recombinant erythropoietin (100 UI/kg SC, three times a week) was implemented when hematocrit was < 15% being associated with orally iron supplementation. After hematocrit returned to the reference interval (30–40%), erythropoietin use was shortened to twice a week [21,22].

Potassium and bicarbonate supplementation was conducted based on serum biochemistry results. If necessary, a maximum of 30 mEq/L of potassium chloride was added to the fluid therapy. Sodium bicarbonate (8 to 12 mg/kg, BID or TID) was orally administered. Systolic blood pressure was controlled with amlodipine (0.1–0.25 mg/kg PO, SID or BID) or benazepril (0.25–0.5 mg/kg PO, SID). Aluminum hydroxide at a daily dose of 30–100 mg/kg was prescribed to control hyperphosphatemia and associated with therapeutic diet for kidney injury patients (Royal Canin Canine Renal Diet, Descalvado, São Paulo, Brazil).

### Intermittent hemodialysis

Hemodialysis sessions were conducted at the Dialysis Center. The equipment used was the 4008F Hemodialysis Machine (Fresenius Medical Care, Bad Homburg Höhe, Germany) with ultrafiltration control, coupled to a reverse osmosis water treatment unit (MCA.OR.PF.01 Palhoça, Santa Catarina, Brazil). The intermittent hemodialysis was performed twice a week, with the first session lasting one hour and the remaining sessions lasting two hours, a total of three or more sessions.

The animals were catheterized with a double lumen catheter (Joline GmbH & Co. Hechingen, Germany) in the right or left jugular, passing in cranial vena cava ending up positioned at the entrance of the right atrium, according to the technique described by Bloom & Labato [3]. A radiographic evaluation was conducted to ensure the correct position of the catheter. The dogs were maintained on an inox table in right or left lateral decubitus and manually restrained, without sedation during extracorporeal dialysis. The dogs in the IHG were submitted to up to seven sessions of dialysis treatment as required for each animal.

The activated clotting time for each animal was hourly measured using an MCA 2000 activated clotting monitor (MCA 2000, Fundação Adib Jatene, São Paulo, Brazil). Heparin sodium (Hemofol®, Cristália Produtos Químicos e Farmacêuticos Ltda, Itapira, São Paulo, Brazil) was used to avoid coagulation, at an initial dosage of 50 UI/kg and interrupted when the clotting time was 1.6 to 2 times higher than the reference interval and extra boluses of heparin were applied if necessary [3].

Capillary hemodialyzers with polysulfone membranes (Fresenius Medical Care, Bad Homburg Höhe, Germany) were selected according to the animal’s weight (0.8 m^2^ dialyzers for animals < 12 kg; 1.5 m^2^ for animals between 12 and 20 kg; and 2.0 m^2^ dialyzers for animals > 30 kg) [23]. The dialysate was comprised by an 8.4% sodium bicarbonate buffer solution (BiBag, Fresenius Medical Care, Jaguariúna, São Paulo, Brazil) and a solution of electrolytes with glucose (CPHD 22G/34 with glucose, Fresenius Medical Care, Jaguariúna, São Paulo, Brazil) to avoid hypoglycemia. The final composition of the dialysate after dilution in ultrapure water obtained from the portable reverse osmosis device (MCA.OR.PF.01, Palhoça, Santa Catarina, Brazil) was 138 mEq/L of sodium, 3 mEq/L of potassium, 35 mEq/L of bicarbonate, 108.5 mEq/L of chloride, 3.5 mEq/L of calcium, and 100 mg/dL of glucose. Sodium was adjusted to 155 mmol/L in the first session and 150 mmol/L in the remaining sessions, aiming to minimize the occurrence of disequilibrium syndrome and low blood pressure [4]. The extracorporeal blood flow was arranged for 5 mL/kg/min for the first session, and then increased to 10 mL/kg/min for the second session and 15 mL/kg/min for the remaining sessions. The dialysate’s flow was 500 mL/min for all animals [4].

### Sample collection and analytical methods

In the CG, blood samples were collected from jugular vein in only one moment in the study. For IHG and CTG, blood samples were collected aseptically from jugular vein 30 minutes before the beginning of the first session of intermittent hemodialysis or clinical treatment (first-moment), and 30 minutes before the beginning of the last session of intermittent hemodialysis or clinical treatment (last-moment).

Red blood cell count was manually performed by using a hemocytometer [24], platelets and leukocytes were counted in an Poch-100iV Diff, while total plasma protein was manually determined by using a hand-held refractometer [15].

Serum concentration of urea, creatinine, albumin, potassium and phosphorus were performed in a Cobas Mira Plus Roche enzyme measurement device according to the manufacturers and commercially available kits’ specifications (Bioclin®, Roche Diagnostic, Belo Horizonte, Minas Gerais, Brazil) [25].

Urine was aseptically collected by cystocentesis in the first and last-moments, and urine protein to creatinine ratio was determined by colorimetric method (Bioclin®, Roche Diagnostic, Rotkreuz, Switzerland) [25].

Serum samples were stored at -80°C for 12 months to determine C-reactive protein concentrations. The concentrations of C-reactive protein were determined by two different commercial kits and used according to manufacturers’ specifications: Dog C-reactive protein ELISA, (Life Diagnostics Inc, West Chester, Pennsylvania, United States) and sandwich ELISA method (Catalyst C-reactive protein test kit, IDEXX Catalyst® device, Westbrook, Maine, United States) [26,27].

**Fig 1:**
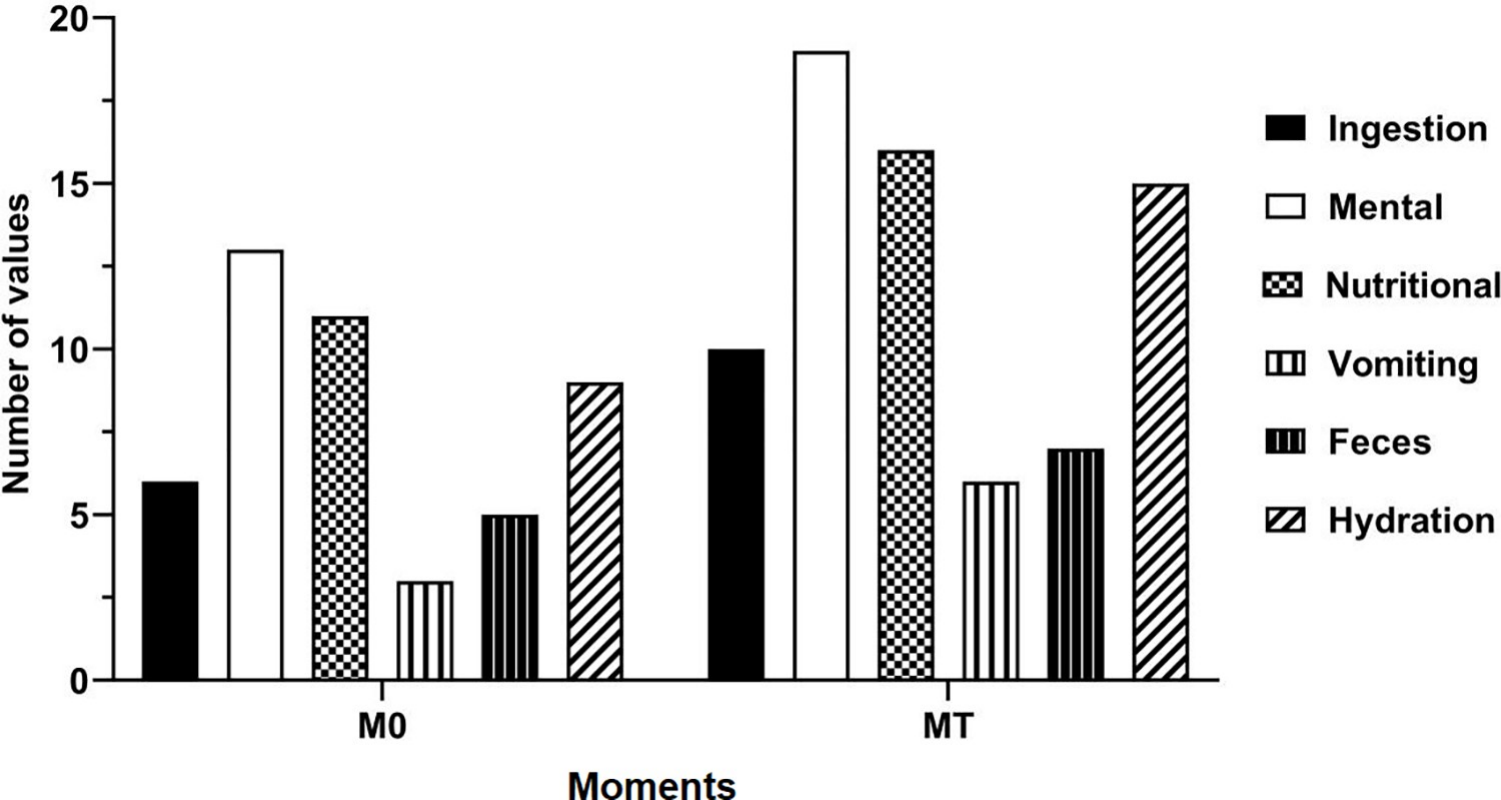
Clinical score plot of dogs with stage IV CKD submitted to intermittent hemodialysis (IHG) at the first- and last-moments.

**Fig 2:**
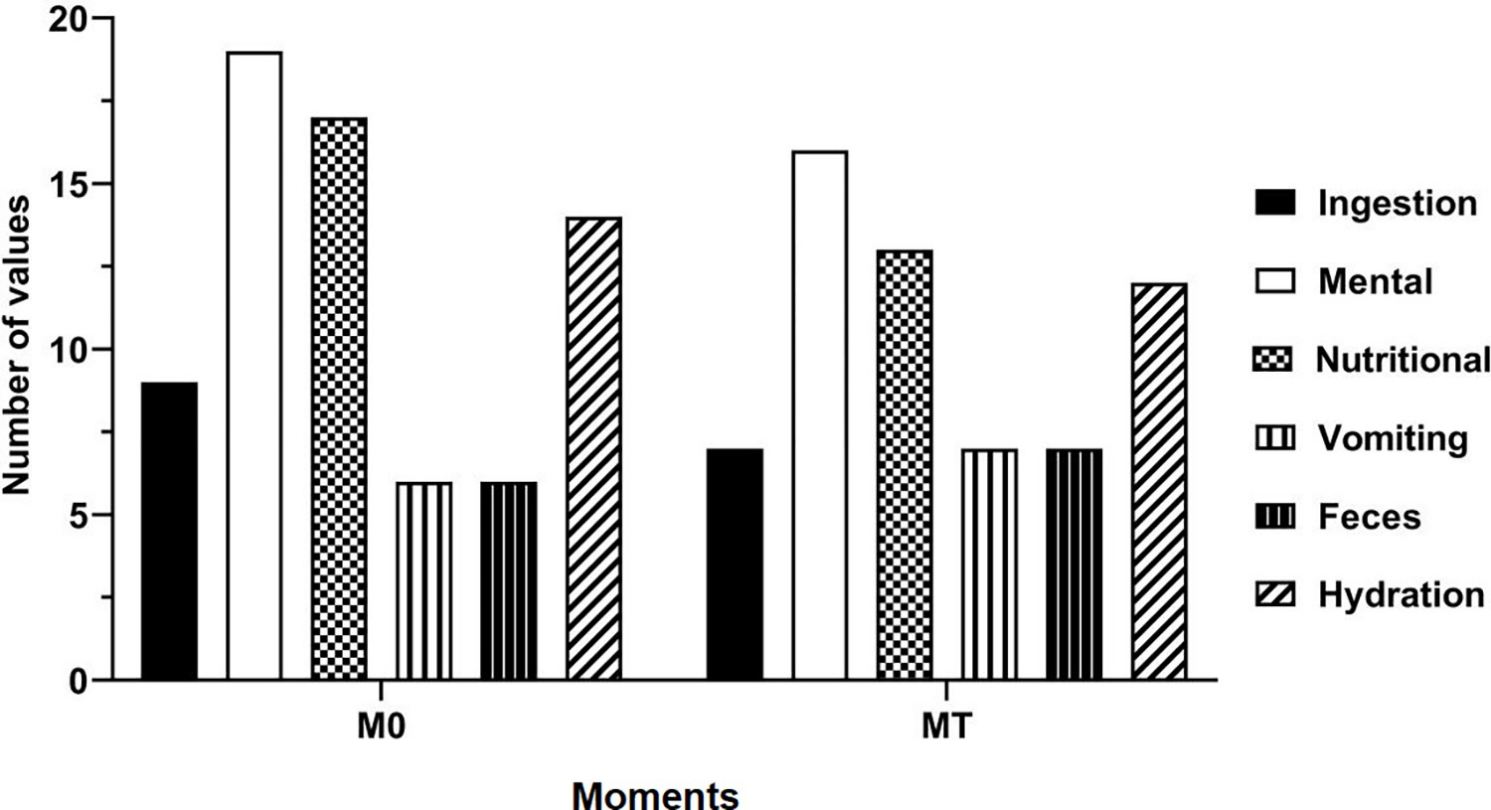
Clinical score plot at the first- and last-moments of clinically treated (CTG) dogs with stage IV CKD.

The clinical score of the patients was also evaluated. The considered criteria for both groups were dehydration, feces, emesis, nutritional and mental status, and dietary intake at the first and last-moments (Figs 1 and 2).

### Statistical analysis

The power of the test was calculated for all variables considering n = 7 (the lower number of dogs/group) and the mean of results was 0.86. The variable “age” for each treatment was determined using Proc Freq. The survival curves for each treatment were calculated using the Kaplan-Meier test. Assumptions regarding normal distribution and homoscedasticity were verified by Shapiro-Wilk and Bartlett tests, respectively. Log-transformation was performed when appliable. The variables from the three groups were compared by ANOVA One Way. Results from the first- and last-moments in the groups and among sessions in the treatment groups were compared with paired t-test for those variables with normal distribution. Wilcoxon and Mann-Whitney tests were used to compare first- and last-moments within a single treatment group and first- or last-moment between treatment groups, respectively. Pearson’s linear correlation analysis was used to assess in the first-moment, the correlation between C-reactive protein, conducted by sandwich ELISA method, and albumin; also the correlation between C-reactive protein by the same method and red blood cell was conducted. Spearman’s linear correlation analysis was used to assess the correlation between C-reactive protein and total leukocytes at the first-moment. Spearman’s correlation analysis was also used to compare both methods (ELISA and sandwich ELISA). The results were presented as correlation coefficients, which were considered to be strong (r > 0.6), moderate (0.6 ≤ r ≥ 0.4), and weak (r < 0.4) [19]. Parametric results were presented as mean ± error standard of the mean, while nonparametric results were presented as median and Q1 and Q3 interquartile. The survival curves for each treatment were calculated using the Kaplan-Meier test. A significance level of 5% was considered for all analyses, which were conducted in GraphPad Prism software version 9.0.2.

## Results

### Clinical findings

A total of 16 dogs were included in the treatment groups (stage IV CKD) and seven in the CG. The IHG was comprised of four mixed-breed, one Basset Hound, one Dalmatian, one Rottweiler and one Border Collie. The CTG was comprised of four mixed-breeds, one American Pit Bull Terrier, one Chow-Chow, one Rottweiler and one Labrador Retriever. The CG was comprised of five mixed-breeds, one Border Collie and one Flat Coated Retriever.

**Fig 3:**
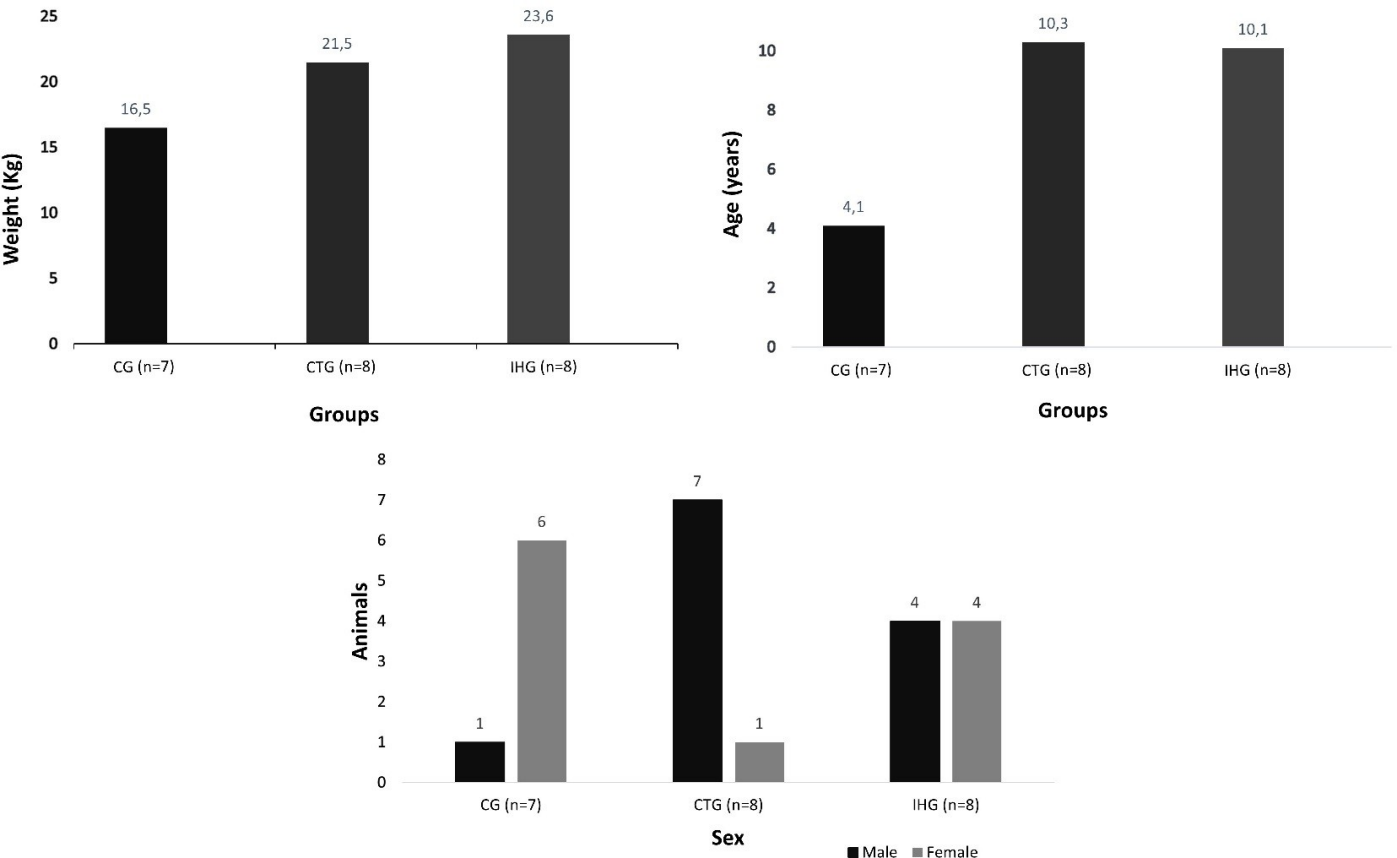
Data on sex and mean of weight and age values of clinically healthy animals (CG) and stage IV CKD dogs treated with intermittent hemodialysis (IHG) and clinical treatment (CTG).

The data on sex, age and weight included in each group are presented in Fig 3.

**Fig 4:**
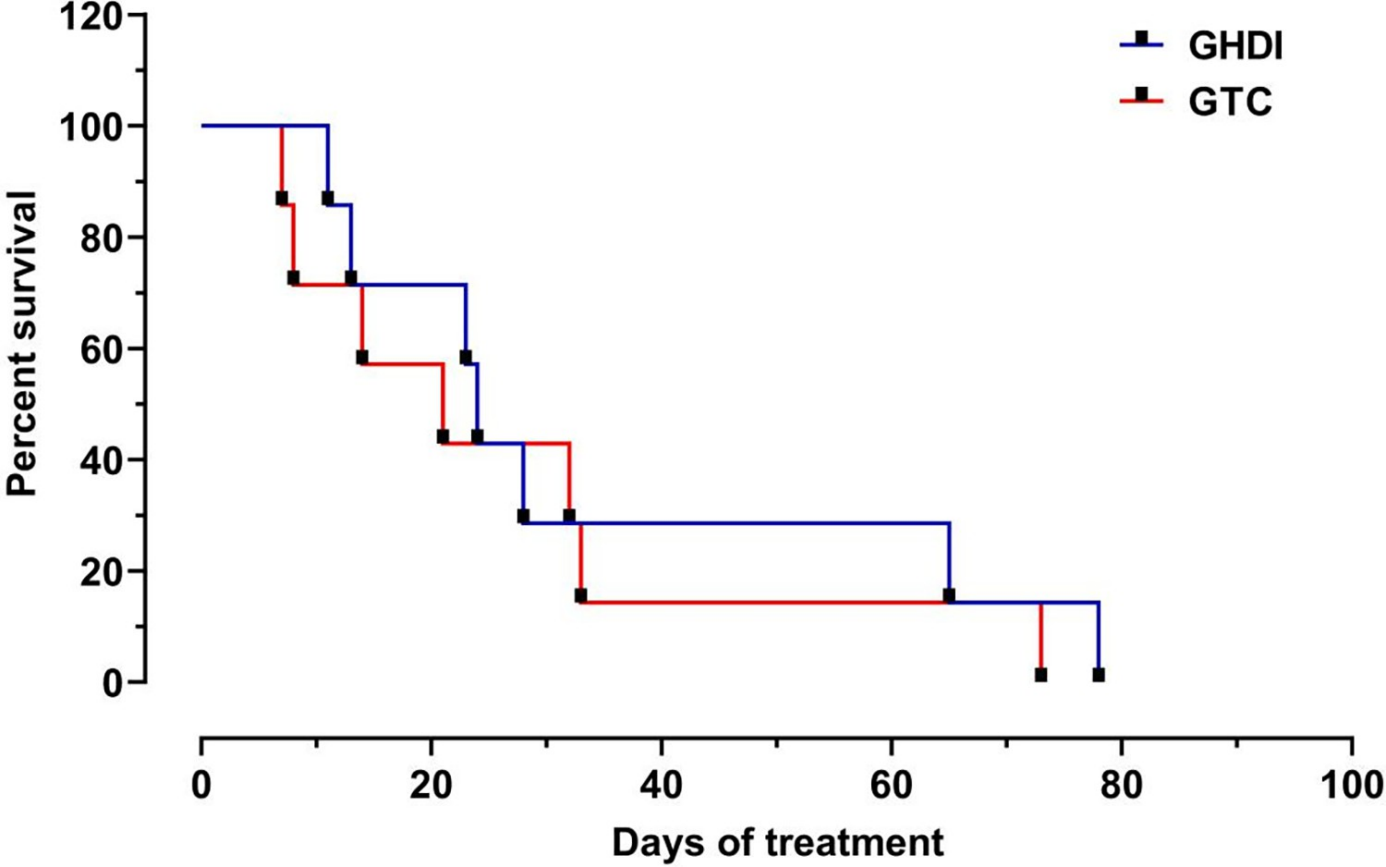
Kaplan-Meyer plot showing the survival rate of dogs with stage IV CKD treated with intermittent hemodialysis (IHG) and clinical treatment (CTG).

All dogs with stage IV CKD died before the end of the study. The animals in the IHG survived approximately 5 days longer than the animals in the CTG, however, there was no significance (P = 0.58) (Fig 4).

### Laboratory findings

C-reactive protein concentrations were different according to the method used (Catalyst® device versus Life Diagnostics®) as there were significance between CG and IHG, both at the first and last-moments (Table 1) for the Catalyst® device. Similarly, there was significance between CTG and IHG at the first-moment for the Life Diagnostics® kit (Table 2).

**Table 1:** Mean ± standard deviation (SD) and confidence interval for the concentrations of C-reactive protein (Catalyst^®^ device), urea (mg/dL), creatinine (mg/dL), phosphorus (mg/dL), and potassium (mEq/L) at the first- and last-moments in dogs from CG, IHG and CTG.

Variables	Moment	CG Mean ± SD (confidence interval)	IHG Mean ± SD (confidence interval)	CTG Mean ± SD (confidence interval)
**C-reactive protein** **(mg/L)**	**First-moment**	3.1 ± 1.9^A^ (1.3–4.9)	45.2 ± 33.3^Ba^ (3.8–86.5)	27.5 ± 18.6^ABa^ (10.3–44.7)
	**Last-moment**	3.1 ± 1.9^A^ (1.3–4.9)	54.5 ± 28.4^Ba^ (30.7–78.2)	35.0 ± 35.7^ABa^ (1.9–68.0)
**Urea** **(mg/dL)**	**First-moment**	43.5 ± 16.1^A^ (28.6–58.4)	337.3 ± 145.8^Ba^ (215.3–459.3)	268.7 ± 87.2^Ba^ (195.7–341.6)
	**Last-moment**	43.5 ± 16.1^A^ (28.6–58.4)	180.0 ± 38.7^Bb^ (147.6–212.4)	334.4 ± 98.8^Ca^ (251.7–417.1)
**Creatinine** **(mg/dL)**	**First-moment**	1.1 ± 0.1^A^ (1.0–1.3)	10.7 ± 5.8^Ba^ (5.8–15.6)	9.0 ± 3.2^Ba^ (6.3–11.7)
	**Last-moment**	1.1 ± 0.1^A^ (1.0–1.3)	7.2 ± 3.0^Bb^ (4.7–9.8)	11.8 ± 5.0^Cb^ (7.6–16.0)
**Phosphorus** **(mg/dL)**	**First-moment**	3.1 ± 0.5^A^ (2.6–3.6)	24.8 ± 22.6^Ba^ (5.9–43.8)	15.9 ± 7.0^ABa^ (10.0–21.8)
	**Last-moment**	3.1 ± 0.5^A^ (2.6–3.6)	12.2 ± 5.5^Ba^ (7.6–16.9)	21.5 ± 8.4^Cb^ (14.4–28.5)
**Potassium** **(mEq/L)**	**First-moment**	3.1 ± 0.2^A^ (2.8–3.3)	3.5 ± 0.6^Aa^ (3.0–4.0)	4.3 ± 0.6^Ba^ (3.7–4.9)
	**Last-moment**	3.1 ± 0.2^A^ (2.8–3.3)	4.5 ± 1.3^Bb^ (3.4–5.6)	4.6 ± 0.9^Ba^ (3.8–5.4)

Different uppercase letters in the same line indicate significance between the groups. Different lowercase letters in the same column indicate significance between the moments. Abbreviations: HIG, intermittent hemodialysis group; CTG, clinical treatment group; CG, control group (healthy animals).

**Table 2:** Median, interquartile interval (25–75%), minimum (min) and maximum values (max) for C-reactive protein (Life Diagnostics^®^), and total leukocytes at the first- and last-moments in dogs from CG, IHG and CTG.

Variables	Moment	CG Median (25–75%) [Min–Max]	IHG Median (25–75%) [Min–Max]	CTG Median (25–75%) [Min–Max]
**C-reactive protein** **(mg/L)**	**First-moment**	44.1^AB^ (40.5–46.6) [36.8–47.1]	66.1^Aa^ (24.2–185.5) [7.8–523.4]	7.8^Ba^ (7.8–36.1) [7.8–232.3]
	**Last-moment**	44.1^A^ (40.5–46.6) [36.8–47.1]	68.2^Aa^ (7.8–88.4) [7.8–122.0]	50.3^Aa^ (40.0–73.2) [37.6–317.1]
**Total leukocytes** **(x10** ^**3**^ **/mm** ^**3**^ **)**	**First-moment**	6.53^A^ (6.1–7.9) [5.5–9.8]	10.45^Ba^ (8.5–10.9) [6.8–15.4]	8.7^ABa^ (6.9–10.8) [5.8–10.8]
	**Last-moment**	6.5^A^ (6.1–7.9) [5.5–9.8]	14.7^Bb^ (11.6–17.2) [11.0–17.5]	8.6^ABa^ (5.9–13.1) [4.6–17.7]

Different uppercase letters in the same line indicate significance between the groups. Different lowercase letters in the same column indicate significance between the moments. Abbreviations: HIG, intermittent hemodialysis group; CTG, clinical treatment group; CG, control group (healthy animals).

Serum C-reactive protein presented a moderate positive correlation with total leukocytes (P = 0.0152), and moderate negative correlation with albumin (P = 0.0069) and red blood cells (P = 0.0131).

**Fig 5:**
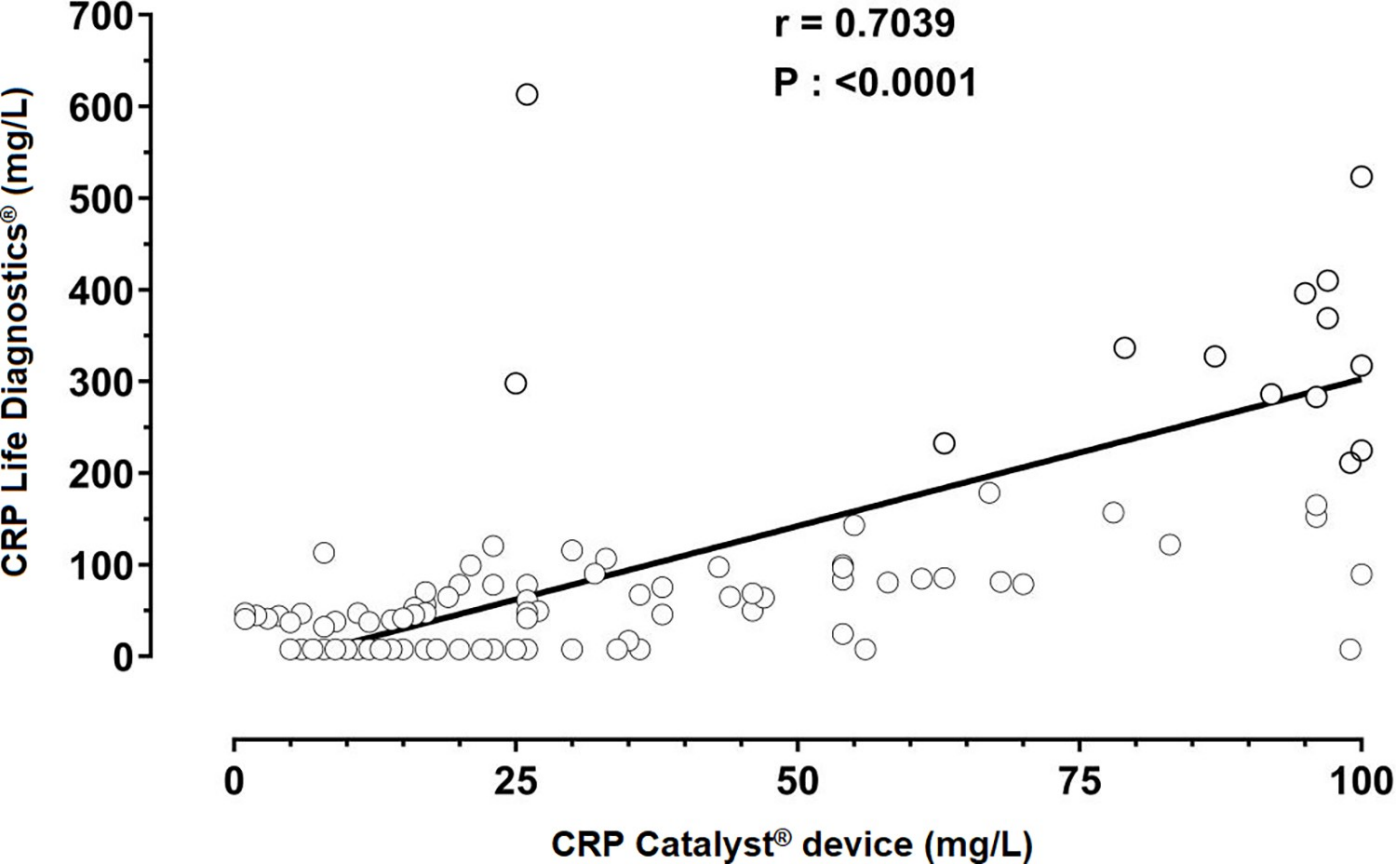
Linear regression plot of the determination of C-reactive protein concentrations in two methods (Life Diagnostics^®^ *versus* Catalyst^®^ device) in stage IV CKD dogs submitted to intermittent hemodialysis and clinical treatment.

A high correlation coefficient (CI = 0.59–0.78; r = 0.70; P < 0.0001) was present in the evaluation of the two tests (Fig 5).

The mean and standard deviation for the concentration of the biochemical variables (urea, creatinine, phosphorus, and potassium), and C-reactive protein (Catalyst® device) are summarized in Table 1. In Table 2 are summarized median, interquartile range, minimum and maximum concentration for C-reactive protein (Life Diagnostics®) and total leukocytes.

In the IHG, urea had a significant reduction (P < 0.001) in the last-moment when compared to CTG (Table 1).

The IHG presented 14.5-fold increase in C-reactive protein concentration (Catalyst® device) at the first-moment and 17.4-fold increase at the last-moment when compared with CG. Additionally, at the first-moment, an increase of 8.4 in C-reactive protein concentration in the IHG compared to the CTG was noted.

## Discussion

Based on the values of C-reactive protein between groups, we observed an active inflammatory condition in patients with stages IV chronic renal failure submitted to intermittent hemodialysis as C-reactive protein concentrations were increased in the IHG when compared to CTG and CG. Indeed, C-reactive protein is produced by hepatocytes in response to pro-inflammatory cytokines, such as interleukin-1, interleukin-6 and tumor necrosis factor alpha [12]. However, C-reactive protein production was also described in the tubular epithelial cells of the renal cortical in humans [28]. Acute phase proteins binds to phosphocholine and recognizes some pathogens and phospholipids in damaged cells [29]. Therefore, this protein plays a role against infections, in the regeneration of damaged tissue, prevention of autoimmunization, and inflammatory responses modulation [30].

In humans, C-reactive protein is used to monitor inflammatory response in patients with CKD [16,31–33]. In veterinary medicine, C-reactive protein concentration increases in response to several conditions [29–32], including naturally-occurring CKD in dogs, in which the activation of acute phase response was associated with the pathogenesis of the disease [19].

In dogs, C-reactive protein is considered a major acute phase protein and may increase 100 to 1,000-fold in animals with systemic inflammation [15]; its concentration varies between 0.44 and 907.4 mg/L [34] in such condition. In our study, high C-reactive protein concentrations in the IHG compared to CG and CTG points that intermittent hemodialysis leads to inflammatory response activation as the interaction between blood and membranes during dialysis, induces to a microinflammation [35]. Therefore, C-reactive protein increased concentration may be a result of the activation of the acute phase response during hemodialysis [32].

The concentrations of C-reactive protein may have been lower than expected for a major positive acute phase protein, as the maximum C-reactive protein expression in the organism could not have been portrayed in the moments that samples collection was made. Nevertheless, similarly to the observed by Raila et al., [19], C-reactive protein concentrations were elevated in CKD dogs. It means that the chronic process can induce to chronic stimulation of the acute phase response, resulting in a chronic inflammatory profile due to systemic alterations related to the disease [8].

We did not observe differences in C-reactive protein concentrations between the first- and last-moments, which can be related to short survival times after the beginning of the treatment. A reduction in protein serum concentration is reported to be observed several months after treatment [33]. The authors associated this reduction to blood purification during dialysis, leading to a reduction of chronic inflammation [33].

The total leukocyte count in the IHG remained within the reference interval for the species, however a significant increase, in the first- and last-moments was observed when compared to CG. In fact, acute phase response stimulation can be associated with the increase of total leukocyte count through the production of inflammatory mediators [17,19,36].

Opposite to our results, Nakamura et al. [17] stated no correlation between C-reactive protein and albumin. Therefore, the negative correlation presented in our study may be related to the production of other acute phase response proteins. This is justified based on the decrease in albumin concentration during inflammatory response [13] and the utilization of amino acids used in albumin synthesis that are also used in positive acute phase proteins production [37].

Inflammation is an important factor associated with anemia in patients with chronic diseases, in which inflammatory cytokines suppress erythropoiesis, lead to iron sequestration, and promote alterations in erythrocytes cell membrane [38]. Both anemia and inflammation are associated with inflammatory markers high concentrations, such as C-reactive protein, which is linked to anemia treatment resistance in CKD patients [38,39]. This may explain the persistence of anemia in CKD patients.

Assays using heterologous anti-CRP antibodies should be validated to measure canine C-reactive protein [40]. The Catalyst CRP test® is an assay that employs mouse IgG anti- C-reactive protein, which may underestimate concentrations [41]. However, the Catalyst® device has been validated and provided a precise quantification of canine serum C-reactive protein [27]. Therefore, the high positive correlation observed between the Catalyst® device and Life Diagnostics® may ensure the confidence to read the results obtained from the assay.

Kidneys are an excretion route for phosphorus and the renal function impairment caused by CKD leads to increased concentrations in blood circulation [42]. This explains the high phosphorus values observed in the IHG and CTG when compared to CG.

Azotemia is an alteration caused by renal function impairment, commonly observed in stage IV CKD patients [4]. This explains the high concentrations of urea and creatinine in the IHG and CTG in comparison to CG at both moments. At the last-moment, IHG presented lower concentrations of urea, creatinine and phosphorus than CTG, which may be explained by the conventional treatment low effectiveness in reducing these catabolites levels [23].

At the beginning of hemodialysis sessions, animals in the IHG presented an unfavorable clinical score. However, after the end of hemodialysis, animals demonstrated a clinical improvement when compared to CTG, confirming the effectiveness of the technique in spite of the existing inflammatory response.

This study demonstrates that stage IV CKD patients submitted to intermittent hemodialysis present an active inflammatory profile, confirmed by C-reactive protein high concentrations measured by using CRP Catalyst® device methodology, which can be used to evaluate the inflammatory activity in this treatment modality. Both assays used in this study to measure C-reactive protein have provided safe and reliable quantification of the results.

Thus, despite the inflammatory reaction caused by both disease and treatment, intermittent hemodialysis has proven to be effective in reducing serum concentrations of urea, creatinine and phosphorus, then leading to a clinical improvement and a better life quality and being recommended for stage IV CKD dogs if performed by trained professionals.

## Supporting information

S1 Dataset (PDF)
