# Supplementary material for: C-reactive protein concentrations are higher in dogs with stage IV chronic kidney disease treated with intermittent hemodialysis
Source: PLoS One. 2022 Sep 22;17(9):e0274510. doi: 10.1371/journal.pone.0274510 (PMC9499245; doi:10.1371/journal.pone.0274510)
Supplement: S1 Dataset — (PDF) [file pone.0274510.s001.pdf]

Values behind the means, standard deviations and other measures reported

Weight, Age, Sex and Survival:

Group	Age (years)	Weight (Kg)	Sex	Survival (days)
**IHG 1**	8	18.7	Male	24
**IHG 2**	13	11.35	Female	23
**IHG 3**	11	21	Female	78
**IHG 4**	9	37	Female	13
**IHG 5**	12	15.5	Male	65
**IHG 6**	7	40.2	Male	78
**IHG 7**	10	21.25	Male	28
**IHG 8**	13		Female	11
**CTG 1**	7	12.6	Female	7
**CTG 2**	12	11.3	Male	73
**CTG 3**	6	16.95	Male	33
**CTG 4**	12		Male	7
**CTG 5**	10	21.8	Male	14
**CTG 6**	7	25.75	Male	2
**CTG 7**	14	25.3	Male	21
**CTG 8**	8	37.5	Male	8
**CG 1**	3	21.15	Female	Not applicable
**CG 2**	6	18.75	Female	
**CG 3**	6	13.65	Male	
**CG 4**	3	25.5	Female	
**CG 5**	1.6	18.25	Female	
**CG 6**	4	17.5	Female	
**CG 7**	5	8.5	Female	

Clinical escore:

Clinical escore	Ingestion		Mental		Nutritional		Vomiting		Feces		Hydration	
**Group**	**First-moment**	**Last-moment**	**First-moment**	**Last-moment**	**First-moment**	**Last-moment**	**First-moment**	**Last-moment**	**First-moment**	**Last-moment**	**First-moment**	**Last-moment**
**IHG 1**	2	2	2	3	2	2	1	1	1	1	2	2
**IHG 2**	0	1	2	2	1	2	1	1	1	1	1	2
**IHG 3**		1		2		2		1		1		2
**IHG 4**	1	1	2	2	3	2	0	1	1	0	2	2
**IHG 5**	1	1	3	3	1	1	0	0	1	1	1	1
**IHG 6**	1	1	2	2	2	3	1	1	0	1	2	2
**IHG 7**	1	2	2	3	2	2	0	1	1	1	1	2
**IHG 8**		1		2		2		0		1		2
**Group**	**First-moment**	**Last-moment**	**First-moment**	**Last-moment**	**First-moment**	**Last-moment**	**First-moment**	**Last-moment**	**First-moment**	**Last-moment**	**First-moment**	**Last-moment**
**CTG 1**	1	1	3	2	2	3	0	1	1	1	2	2
**CTG 2**	1	1	3	2	2	2	1	1	1	1	2	2
**CTG 3**	1	1	2	2	2	1	0	0	1	1	2	1
**CTG 4**	1	0	2	1	2	1	1	1	1	1	1	1
**CTG 5**	1	0	2	2	3	1	1	1	0	0	2	1
**CTG 6**	2	2	3	3	3	2	1	1	1	1	2	2
**CTG 7**	1	1	1	2	1	1	1	1	1	1	1	1
**CTG 8**	1	1	3	2	2	2	1	1	0	1	2	2
**CG**	Not applicable											

Laboratorial parameters - Intermittent hemodialysis group (IHG)

First-moment

First-moment	C-reactive protein (Catalyst® device) (mg/L)	C-reactive protein (Life Diagnostics®) (mg/L)	Urea (mg/dL)	Creatinine (mg/dL)	Serum albumin (g/dL)	Phosphorus (mg/dL)
**IHG 1**	26	61.3	283	5.1	1.8	18
**IHG 2**	25	7.8	269	6.4	2.9	31.9
**IHG 3**	100	523.45	254	11.5	2.4	13.1
**IHG 4**		24.65	171	6.6	2.9	2.7
**IHG 5**	54	24.15	236	6.45	2.2	11
**IHG 6**	21	99.2	381	11.26	1.8	75.5
**IHG 7**		214.2	521	16.72	2.4	15.8
**IHG 8**		71	584	21.6	2.3	31

First-moment	Potassium (mEq/L)	Erythrocytes (/µL)	Total plasma protein (g/dL)	Platelets (/µL)	Total leukocytes (/µL)	Urinary protein to creatinine ratio
**IHG 1**	4	2.81	9	224.000	8.400	7.2
**IHG 2**	2.9	6.72	6.4	118.000	6.800	4.3
**IHG 3**	3.9	4.3	7.4	186.850	15.400	2.6
**IHG 4**	3.4	5.47	7.6	383.800	10.700	1.6
**IHG 5**	4.1	4.65	9	297.000	10.800	1.8
**IHG 6**	4.3	2.3	5.4	162.000	8.800	1.8
**IHG 7**	3.4	2.93	7	250.000	10.900	1.4
**IHG 8**	2.4	2.4	9.8	146.450	10.200	1.3

Laboratorial parameters - Clinical treatment group (CTG)

First-moment

First-moment	C-reactive protein (Catalyst® device) (mg/L)	C-reactive protein (Life Diagnostics®) (mg/L)	Urea (mg/dL)	Creatinine (mg/dL)	Serum albumin (g/dL)	Phosphorus (mg/dL)
**CTG 1**	25	7.8	177	5.8	2.9	26.9
**CTG 2**	11	7.8	291	9.27	2.9	9.4
**CTG 3**	63	232.35	232.4	5.7	2.6	13
**CTG 4**	30	7.8	417	9.48	2.7	26
**CTG 5**	12	7.8	269.8	13.99	3.4	17.24
**CTG 6**	14	7.8	158	5.2	2.3	8.35
**CTG 7**	46	7.8	359.4	11.83		14.63
**CTG 8**	38	45.6	245	10.99	2.3	12.29

First-moment	Potassium (mEq/L)	Erythrocytes (/µL)	Total plasma protein (g/dL)	Platelets (/µL)	Total leukocytes (/µL)	Urinary protein to creatinine ratio
**CTG 1**	4.3	2.35	7	318.000	10.800	7.26
**CTG 2**	4.2	4.99	7.2	514.000	7.100	1.9
**CTG 3**	3.7	6.61	8	277.000	5.800	3.22
**CTG 4**	3.7	4.29	10.2	308.050	10.800	1.85
**CTG 5**	4.5	3.86	8	358.000	6.900	2.21
**CTG 6**	4.1	4.51	7.8	187.000	8.700	2.62
**CTG 7**	4.6					0.77
**CTG 8**	5.9	2.61	6.8	323.500	9.700	3.14

Laboratorial parameters - Control group (CG)

First-moment

First-moment	C-reactive protein (Catalyst® device) (mg/L)	C-reactive protein (Life Diagnostics®) (mg/L)	Urea (mg/dL)	Creatinine (mg/dL)	Serum albumin (g/dL)	Phosphorus (mg/dL)
**CG 1**	4	44.15	31	1.11	3.7	3.2
**CG 2**	3	41.15	43	1.24	3.6	3
**CG 3**	2	44.35	28	1.4	3.4	2.1
**CG 4**	1	47.15	39	1.13	4.1	3.3
**CG 5**	6	46.65	53	1.21	3.5	3.2
**CG 6**	5	36.85	75	1.21	3.5	3.1
**CG 7**	1	40.55	36	0.95	3.9	3.9

First-moment	Potassium (mEq/L)	Erythrocytes (/µL)	Total plasma protein (g/dL)	Platelets (/µL)	Total leukocytes (/µL)	Urinary protein to creatinine ratio
**CG 1**	2.9	7.170	6.2	227.000	6.530	0.06
**CG 2**	3	6.290	7.2		7.870	0.07
**CG 3**	3.1	7.400	7	173.000	5.500	0.05
**CG 4**	2.8	7.860	8.4	257.000	6.100	0.17
**CG 5**	3.2		6.2	196.950	9.800	0.07
**CG 6**	3.2		6.6	170.000	6.300	0.09
**CG 7**	3.5	6.880	7.2	338.000	7.300	0.2

Laboratorial parameters - Intermittent hemodialysis group (IHG)

Last-moment

Last-moment	C-reactive protein (Catalyst® device) (mg/L)	C-reactive protein (Life Diagnostics®) (mg/L)	Urea (mg/dL)	Creatinine (mg/dL)	Serum albumin (g/dL)	Phosphorus (mg/dL)
**IHG 1**	56	7.8	177	6.3	2	15.2
**IHG 2**	25	7.8	208	4.5	2.6	21.8
**IHG 3**	83	122	202	6.1	2.6	10.4
**IHG 4**	58	80.5	143	6.9	2.6	5.4
**IHG 5**	17	55.9	130	3.74	2.5	5.3
**IHG 6**	142	89.5	179	7.4	2.7	12
**IHG 7**	34	7.8	153	11.02	2.9	11.5
**IHG 8**	63	85.25	248.2	12.43	2.5	16.42

Last-moment	Potassium (mEq/L)	Erythrocytes (/µL)	Total plasma protein (g/dL)	Platelets (/µL)	Total leukocytes (/µL)	Urinary protein to creatinine ratio
**IHG 1**	6	2.79	8.6	244.000	17.500	7.9
**IHG 2**	3.1	3.1	5.4	389.000	12.700	7.8
**IHG 3**	4.1	3.42	6.6	290.000	11.500	1
**IHG 4**	3.6	4.3	7.4	383.000	17.200	1
**IHG 5**	3.9	3.71	7.4	302.000	11.800	0.7
**IHG 6**	7	3.38	7.8	153.000	16.700	4.9
**IHG 7**	4.8	2.72	7	234.000	10.990	1.6
**IHG 8**	4	2.35	9.4	231.000	17.100	1.45

Laboratorial parameters - Clinical treatment group (CTG)

Last-moment

Last-moment	C-reactive protein (Catalyst® device) (mg/L)	C-reactive protein (Life Diagnostics®) (mg/L)	Urea (mg/dL)	Creatinine (mg/dL)	Serum albumin (g/dL)	Phosphorus (mg/dL)
**CTG 1**	9	37.6	218	9.3	2.9	32.6
**CTG 2**	27	49.2	356.3	9.39	2.7	15
**CTG 3**		47.9	322.6	8.1	2.8	25
**CTG 4**	167	317.1	527	13.05	2.5	29
**CTG 5**	8	32.05	384.9	20.72	2.9	29.59
**CTG 6**	16	52.85	217.9	6.7	2.1	11.87
**CTG 7**	70	78.75	345.1	9.66	2.6	12.45
**CTG 8**	15	41.5	303.7	18.08	2.4	16.62

Last-moment	Potassium (mEq/L)	Erythrocytes (/µL)	Total plasma protein (g/dL)	Platelets (/µL)	Total leukocytes (/µL)	Urinary protein to creatinine ratio
**CTG 1**	4.3	3.53	7	434.300	8.600	5.7
**CTG 2**	4.1	3.64	7	441.000	7.200	2.5
**CTG 3**	3.7	7.4	8	331.000	4.600	2.5
**CTG 4**	4.1	3.94	9.6	346.000	17.700	1.6
**CTG 5**	5.1	3.35	6.4	179.000	11.200	1.41
**CTG 6**	4.1	4.52	7.8	187.000	5.900	3.27
**CTG 7**	5.3	3.54	8.4	98.000	12.700	3.65
**CTG 8**	6.6	2.37	6.8	372.000	13.100	1.9

Laboratorial parameters - Control group (CG)

Last-moment

Last-moment	C-reactive protein (Catalyst® device) (mg/L)	C-reactive protein (Life Diagnostics®) (mg/L)	Urea (mg/dL)	Creatinine (mg/dL)	Serum albumin (g/dL)	Phosphorus (mg/dL)
**CG 1**	4	44.15	31	1.11	3.7	3.2
**CG 2**	3	41.15	43	1.24	3.6	3
**CG 3**	2	44.35	28	1.4	3.4	2.1
**CG 4**	1	47.15	39	1.13	4.1	3.3
**CG 5**	6	46.65	53	1.21	3.5	3.2
**CG 6**	5	36.85	75	1.21	3.5	3.1
**CG 7**	1	40.55	36	0.95	3.9	3.9

Last-moment	Potassium (mEq/L)	Erythrocytes (/µL)	Total plasma protein (g/dL)	Platelets (/µL)	Total leukocytes (/µL)	Urinary protein to creatinine ratio
**CG 1**	2.9	7.170	6.2	227.000	6.530	0.06
**CG 2**	3	6.290	7.2		7.870	0.07
**CG 3**	3.1	7.400	7	173.000	5.500	0.05
**CG 4**	2.8	7.860	8.4	257.000	6.100	0.17
**CG 5**	3.2		6.2	196.950	9.800	0.07
**CG 6**	3.2		6.6	170.000	6.300	0.09
**CG 7**	3.5	6.880	7.2	338.000	7.300	0.2
